# Language and sound exposure across neonatal intensive care hospitalization and relationships with early outcome

**DOI:** 10.1038/s41372-026-02623-y

**Published:** 2026-03-13

**Authors:** R. Pineda, LJ Woodward, ZA Vesoulis, CD Smyser, TE Inder, AM Mathur, BL Schlaggar

**Affiliations:** 1 Chan Division of Occupational Science and Occupational Therapy, University of Southern California, Los Angeles, CA, USA.; 2 Department of Pediatrics, Keck School of Medicine, Los Angeles, CA, USA.; 3 Gehr Family Center for Health Systems Science and Innovation, University of Southern California, Los Angeles, CA, USA.; 4 Program in Occupational Therapy, Washington University, St. Louis, MO, USA.; 5 Faculty of Health, University of Canterbury, Christchurch, New Zealand.; 6 Department of Neurology, Pediatrics, Radiology, Psychiatry, and Neurosurgery, Washington University, St. Louis, MO, USA.; 7 Center for Perinatal and Infant Research, Children’s Hospital of Orange County, Department of Pediatrics, University of California, Irvine, CA, USA.; 8 Department of Pediatrics, St. Louis University, St. Louis, MO, USA.; 9 Kennedy Krieger Institute, Baltimore, MD, USA.; 10 Departments of Neurology and Pediatrics, Johns Hopkins University School of Medicine, Baltimore, MD, USA.

## Abstract

**OBJECTIVES:**

Examine relationships between quantitative measures of language and sound exposure in the NICU and infant neurobehavior.

**STUDY DESIGN:**

Sixty-four preterm infants (≤28 weeks of gestation) had language and sound exposure measured across four time points: within two weeks of birth and at 30, 34, and 35–41 weeks postmenstrual age (PMA). Neurobehavior was assessed at 35–41 weeks PMA using the NICU Network Neurobehavioral Scales.

**RESULTS:**

Higher average decibel levels in the NICU environment were associated with lower infant orientation scores (*p* = 0.04, β = −0.33). Higher peak decibel levels were associated with greater hypertonia (*p* = 0.01, β = 0.37). More electronic sound exposure was associated with less infant hypotonia (*p* = 0.047; β = −0.003). Increased silence was associated with greater infant hypertonia (*p* = 0.01; β = 0.001). Higher adult word counts were related to lower infant stress (*p* = 0.045, β = −1.37).

**CONCLUSION:**

NICU sound exposures were related to neonatal neurobehavior near term age, highlighting the neurological impact of the auditory environment on preterm infants.

## INTRODUCTION

Each year, approximately 370,000 infants are born preterm in the United States alone [1]. Advanced medical technologies have improved infant survival rates at increasingly lower gestational ages. However, risks for long-term morbidities remain high, with lifelong motor and psychological impacts throughout the lifespan among infants born very preterm (VPT; <32 weeks gestation) [2]. The risk of developmental impairments increases as the estimated gestational age (EGA) at birth decreases [3, 4]. Language and communication problems emerge early and are common, affecting around 30% of VPT children [5, 6]. Furthermore, these problems persist into childhood [7] and often adversely impact other areas, including emotional and behavioral regulation, school achievement, and social relationships [8–11]. Given that infants born VPT spend extended time in the neonatal intensive care unit (NICU) during a period of rapid brain development, modifiable factors within this early environment are receiving increased attention.

A growing body of MRI studies has demonstrated the dramatic structural and functional changes that occur in the developing brain during the third trimester of pregnancy. These include cortical folding and the formation of complex cortical networks critical for future functioning, including behavior, cognition, and language [12, 13]. Further, there are marked developmental changes evident in the auditory system from preterm birth until term-equivalent age, the time the infant would have been born had they not been preterm. The auditory system completes the connection between the cochlea and the brainstem by 24–25 weeks of gestation, and to the temporal lobe (including auditory cortex) as early as 30–31 weeks of gestation [14]. Auditory evoked potentials are evident by the 28th week, and from 28 to 34 weeks of gestation, there is a marked reduction in response latency over this period, which is believed to result from maturing brainstem conduction and improved synaptic efficiency [15]. However, for the infant born VPT, these changes are occurring in the ex-utero environment of the NICU [16, 17], which may alter the developmental trajectory.

Prior to full-term birth, infants have the capacity for auditory responses, and it is believed that the pitch, intensity, and pattern of in utero auditory exposures serve to stimulate the system and foster connections between the cortex and the cochlea. In utero auditory exposure, in which high-frequency noise is filtered out, is understood to facilitate the acquisition of speech and language, with human speech perceived and learned by the fetus between 31 and 40 weeks of gestation [18]. Supporting this notion, the two-day-old newborn demonstrates a preference for the mother’s voice [19]. Mounting evidence suggests that hearing the mother’s voice prior to birth plays an important role in experience-dependent brain and behavioral development [19, 20], while other studies suggest that the timed auditory input of low-frequency sounds as experienced in utero, followed by later sounds at higher frequencies, is important for normal auditory development [21]. For example, research conducted in deaf populations shows the arrest of auditory cortex development during periods of deafness [22]. However, it remains unclear how the altered auditory environment of the NICU independently inﬂuences the auditory and neurobehavioral development of the VPT infant.

Previous research has demonstrated the feasibility of using the Language and ENvironment Analysis (LENA) device in VPT infants in the NICU to capture and quantify the amount of real-time language and sound exposures, including conversational turns (back-and-forth communications between an infant and others in their environment). Our previous work has shown that language exposure (specifically language, word count, and electronic sounds) increased across postmenstrual age (PMA) during NICU hospitalization, that different NICU environments pose different auditory experiences (more silence in private rooms), and that silence increased while noise decreased across PMA [23]. However, therapeutic auditory exposures, such as language and music, are different from the potentially disruptive sounds of the alarms and machines that make up much of the NICU auditory environment.

The period in the NICU for a VPT infant prior to term appears to be a critical period, inﬂuenced by human language exposure [24, 25]. Infants of mothers who experience stress with subsequent decreased verbal engagement with their children, in addition to infants hospitalized in the NICU, have been shown to have poorer language skills in early childhood [26]. A higher level of language exposure in the NICU has been linked to more infant vocalizations and conversational turns [27]. However, therapeutic auditory interventions must be balanced with neuroprotection aimed at minimizing loud and harmful auditory stimuli in the NICU environment. Sound levels have been consistently reported to exceed American Academy of Pediatrics guidelines across NICU settings and can negatively impact infant physiology [28]. Based on principles of developmental care aimed at reducing adverse harsh auditory sensory exposures, it is possible to also inadvertently reduce or eliminate positive sounds, such as human language exposure, that appear important for typical brain development. While the use of single-patient rooms can reduce or eliminate noxious auditory exposures, the transition to the use of private rooms in the NICU has also been found to potentially reduce the amount of environmental enrichment available to an infant [29]. Although family-centered care is common in the NICU [30], many parents are not able to be present and/or engaged in the care of their hospitalized infant [31]. Culture, as well as organizational and individual characteristics, may drive family participation in the NICU, resulting in different auditory environments and contributing to subsequent outcomes. By understanding auditory exposures and how they relate to infant outcomes, we can better target the optimal auditory environment for VPT infants.

The objective of this study was to assess the relationships between quantitative measures of language and sound exposure during the NICU stay and infant neurobehavioral outcomes at term-equivalent age in a cohort of infants born VPT (>28 weeks EGA).

## METHODS

This prospective observational study was approved by the Washington University School of Medicine Institutional Review Board (IRB), and written parental consent was obtained. This study was also approved by the IRB at the University of Southern California.

### Participants and study site

Participants included consecutive admissions to an 85-bed Level IV NICU consisting of beds within a single-patient room or open ward. Exclusion criteria included a known congenital anomaly and a failed hearing test. Over the course of the study, 64 infants born VPT (≤28 weeks EGA) were enrolled within the first 24 h of birth and assessed until term-equivalent age (35–41 weeks PMA).

### Parent and infant characteristics

Infant clinical and family background measures were collected from the electronic medical record. Infant measures included sex, EGA at birth, birth weight, delivery type (Cesarean or vaginal), Apgar scores at 1 and 5 min, Clinical Risk Index for Babies (CRIB) score [32], length of stay, days on total parenteral nutrition, days of breast milk feeding, days of high-frequency oscillatory ventilation and endotracheal intubation, days on continuous positive airway pressure support, days on a nasal cannula, and total hours of oxygen delivery. In addition, NICU room type (private room or open ward) and a range of medical risks were recorded, including grade III or IV intraventricular hemorrhage from cranial ultrasound and/or MRI, patent ductus arteriosus (requiring indomethacin or surgical ligation), necrotizing enterocolitis (stage IIb or greater), and retinopathy of prematurity (requiring surgical intervention). Parental and family factors assessed included infant race (African-American/Black or not African-American/Black), maternal age, marital status, insurance type (public or private), and whether there was maternal prenatal drug use (nicotine, alcohol, and illicit drugs).

### Overview of study procedures

Language and sound exposure in the NICU were captured using the LENA device during four separate 16-h periods over the course of each infant’s NICU hospitalization. Neurobehavioral performance was assessed between 35 and 41 weeks PMA using the NICU Network Neurobehavioral Scales (NNNS) [33].

### Sound and language exposure

At four time points across each infant’s NICU stay [within 2 weeks of birth; 30 weeks PMA (±1week); 34 weeks PMA (±1week); and between 35 and 41 weeks], a 16-h auditory sample was collected using the LENA device [34] placed within two feet of the infant’s ear and secured to the incubator or crib. Auditory data at each time point were downloaded into the LENA Pro software, which generated an estimate of adult words spoken to and around the infant, as well as quantified other environmental noise, including the amount of silence, electronic sounds, meaningful and distant language, and the highest and average decibel levels. Previous research has been conducted on infants in the NICU using the LENA at 32 weeks and 36 weeks PMA, making it a feasible method to capture noise and language in the NICU environment [27, 35–37]. The LENA device has demonstrated robust reliability and validity [38].

### Infant neurobehavior prior to NICU discharge (35–41 weeks PMA)

The NNNS was administered by a trained examiner blinded to the infant’s neonatal course at hospital discharge (range: 35–41 weeks PMA) [33]. The NNNS is a comprehensive 115-item evaluation of infant behavior, self-regulation, and reﬂexes and responses, which takes 20–25 min to conduct at the infant’s bedside. From this assessment, 13 summary scores are generated: Habituation, Orientation, Excitability, Lethargy, Stress, Sub-optimal Reﬂexes, Asymmetry, Quality of Movement, Hypertonia, Hypotonia, Arousal, Handling, and Self-Regulation. Habituation was not scored in this cohort due to the need for a quiet environment. The NNNS has a training program that ensures adequate interrater reliability (>80% agreement). The NNNS has established predictive validity and acceptable internal consistency with Cronbach’s alpha between 0.87 and 0.90. The NNNS has been shown to be predictive of later childhood outcomes, including motor function, cognition, and behavioral outcomes [39–42].

### Statistical analysis

Descriptive statistics were used to describe parent and infant characteristics of the sample as well as neurobehavioral outcomes of the cohort.

The average adult words spoken per day and the amount of environmental noise (percentage of time with noise, silence, electronic sounds, meaningful language, distant language, peak decibel level, and average decibel level) were independent variables used to investigate the relationship of sound to outcome. Given the repeated measures for the sound exposures across the four time points, an area under the curve (AUC) summary statistic was calculated for each sound exposure. The AUC is a method to quantify the total value of a changing variable by integrating (summing) the area under the curve of sound exposure (y-axis) over time (x-axis). The AUC best represents the total accumulated value (or magnitude) across each independent variable. The trapezoidal method was used to calculate the AUC for each sound exposure. The AUC was further divided by the number of weeks between the first measurement (EGA+1 to represent the average timing of the first measurement) and 38 weeks PMA (the estimated average age of the final measurement), to provide an AUC interpretation on a weekly scale. The main analysis involved all infants who had recordings across each time point. However, the AUC was also estimated for infants with fewer than four recordings, according to the time across recordings, and analyses were re-run with this additional data.

Summary scores from the NNNS assessment conducted near term were used as dependent variables, providing measures of infant Orientation, Handling, Quality of Movement, Self-regulation, Stress, Arousal, Sub-optimal Reﬂexes, Hypertonicity, Hypotonicity, Asymmetry, Excitability, and Lethargy. Habituation was not scored due to environmental noise that could impact this outcome.

Associations between AUC values for language and sound exposure (from the LENA device) and infant neurobehavioral (NNNS) outcomes at term age (35–41 weeks PMA) were examined using linear regression models. All associations were adjusted for PMA at the time of testing, given previous studies demonstrating that neurobehavior changes across PMA [16].

To determine the multivariate model, infant and parent factors were investigated for their relationships with language exposure (specifically, the number of adult words), as well as neurobehavior (specifically, the Stress score on the NNNS) using linear regression models. Those factors related to both sound exposure and neurobehavior (*p* < 0.05) were considered for inclusion in the multivariate statistical model. All factors considered for inclusion in the multivariate model were evaluated for collinearity. When variables were relatively highly correlated (*p* < 0.05; *r* > 0.30), there was careful consideration of which variable to include in the statistical model, with a plan to choose the one that was believed to best represent the factor that impacted the outcome. Associations between language and sound exposure and neurobehavior were then explored using multivariate regression models, controlling for such factors.

## RESULTS

Sixty-four infants were enrolled. Ten (16%) expired prior to discharge, but all had at least one recording. Of the remaining 54 infants, all had recordings across all time points, with the exception of four infants with a missing recording at birth (due to inability to capture in time, LENA malfunction, or birth at 28 weeks EGA resulting in only a 30 week PMA recording); none missed a recording at 30 weeks PMA or 34 weeks PMA; and six missed a recording at term age (due to early discharge, infant being too sick, or LENA malfunction). There were 44 infants who had neurobehavioral assessments between 35 and 41 weeks PMA. The infant clinical and family background characteristics of these infants are summarized in Table 1.

All sound recordings were 16 h in duration. Table 2 shows the range and mean (±standard deviation) of meaningful language, number of adult words, electronic noise, and silence averaged across the four assessment time points. Meaningful language, number of adult words, electronic noise, and silence increased across PMA. Whereas the amount of noise exposure decreased across PMA. The average sound levels in the NICU were 58.9 (±3.6) decibels, with an average peak level of 86.9 (±1.4) decibels.

See Table 3 for outcomes of the sample between 35 and 41 weeks PMA. The normative values for each NNNS summary score for a 1–2-day-old, full-term, and healthy sample [43] are also reported for comparison purposes.

To determine the multivariate model, we found that factors related to adult word count included infant sex (*p* = 0.02), CRIB score (*p* < 0.001), EGA (p = 0.003), and birth weight (*p* < 0.001). However, none of these factors were related to the NNNS Stress scores (*p* > 0.05). Therefore, only univariate analyses are reported (with the exception of controlling for PMA at the time of the assessment conducted between 35 and 41 weeks of PMA).

Table 4 describes the associations between the different types of sound exposure over each infant’s NICU stay and neurobehavioral outcomes near term equivalent. Results show that higher average sound decibel levels were related to lower infant orientation scores (*p* = 0.04, β = −0.33), and higher peak sound decibel levels were related to more infant hypertonia (*p* = 0.01, β = 0.37). Greater electronic sounds were related to less infant hypotonia (*p* = 0.047; β = −0.003). More silence was related to greater infant hypertonia (*p* = 0.01; β = 0.001). Greater adult word counts were related to less infant stress (*p* = 0.045, β = −1.37). There were no other relationships between sound exposure and infant neurobehavioral measures. When including all data available, including those with a missing recording, these findings remained largely unchanged.

## DISCUSSION

The key findings of this study suggest that higher levels of language exposure (number of words spoken around the infant) over the course of the infant’s NICU stay were associated with lower levels of infant stress at the time of hospital discharge, while higher intensity of background and unnatural sounds were associated with more adverse infant orientation and motor tonal responses. However, longer exposure to silence was also related to tonal abnormalities. Further, higher levels of electronic sounds, which could include the sound of monitors or music being played, were related to less hypotonia. These findings emphasize the neurological and developmental impact of the auditory environment in the NICU on the VPT infant.

We have previously identified that across PMA, meaningful language, the number of adult words, and electronic sounds increase within the NICU setting [44]. The type of NICU environment (open ward compared to private room) and whether the parents are present also impact the auditory environment [44]. Further, levels of language exposure in the NICU are significantly lower than those experienced in utero by fetuses or by healthy newborns during the same developmental stage [45, 46]. As large between-infant variability in auditory exposure in the NICU has been reported [47, 48], opportunities to quantify and then modify language exposure may aid in optimizing neurobehavioral and neurodevelopmental outcomes in preterm infants. However, studies with direct linkage between the type and quantity of auditory exposures in the NICU and infant outcomes remain limited.

Our study also identified relationships between higher average decibel levels and decreased infant orientation. Orientation refers to the quality of an infant’s visual and auditory attention to a human face and voice, as well as to inanimate objects (such as a rattle and red ball). It is well-understood that infants demonstrate an arousal and orienting response to auditory stimuli [49–52]. However, young infants demonstrate the ability to habituate to stimuli, enabling them to disengage and rest [53, 54]. It is also plausible that infants who are regularly exposed to loud/higher sounds may demonstrate the need for more intense stimuli to orient. This, in turn, could have resulted in poorer orientation responses in infants exposed to routinely higher decibel levels within the NICU environment. Orientation is an important component of social interaction, impacting reciprocal responses with their caregivers, which further drives brain development. Our findings are consistent with other literature that has demonstrated that higher sound levels have negative effects on the developing brain [55].

Adverse relationships were also found between higher peak decibel levels and alterations in infant tone. While causal relationships cannot be established, it is unclear whether there could be an impact of high decibel stimuli, or if increased medical complexity, which is associated with more neurodevelopmental impairment, could have resulted in higher decibel stimuli in the NICU environment. The latter would be consistent with existing research showing that preterm infants who are concurrently on mechanical ventilation, have more pumps present at bedside, or are in an incubator are exposed to higher decibel stimuli [23]. Physiological sequelae have been reported in the NICU when sound exceeds 70 decibels, and loud transient noises impact the cardiovascular and respiratory systems [55, 56]. While immediate effects could potentially be observed on tonal responses, perhaps longer-term consequences can occur based on the frequency and length of time of these physiological responses. Alternatively, infants with higher medical complexity and thus at higher risk of neurodevelopmental impairment are more likely to have intense medical interventions, such as advanced respiratory support. Medical interventions, such as high-frequency oscillatory ventilation, have been reported to increase the intensity of sound within the NICU environment [44]. Such equipment also has embedded alarm systems that could result in higher peak decibel stimuli. Furthermore, the Joint Commission on Accreditation of Healthcare Organizations, in conjunction with the International Electrotechnical Commission, mandates certain alarm volume parameters in the hospital setting, based on an adult medicine context. Such rules can inﬂuence the overall NICU sound environment and may justify careful future consideration of alternatives, given the fragile period of development for infants in the NICU.

While studies of sound reduction programs through the use of infant earmuffs have demonstrated favorable infant outcomes, perhaps suggesting a role for sound exposure limits [57–59], caution is likely indicated on the other end of the spectrum related to silence. We identified a relationship between silence and hypertonia. This relationship is also likely contextual. For example, a plausible explanation may be that greater silence in the NICU may reﬂect lower levels of infant stimulation and lower presence of parents and/or staff. Research in the NICU has confirmed that gentle positive stimuli (such as kangaroo care, hand hugs, massage, and movement) relate to better outcomes [60, 61]. When human interaction, such as during these interventions, does not occur, silence likely replaces it. Resultant hypertonia might then be observed in the infant if they receive less holding and/or activity during the silence, with more time spent lying in their incubator or crib. However, our findings must be carefully considered in that higher decibel stimuli and electronic sounds were also associated with tonal abnormality, making careful attention to positive and appropriate sounds warranted.

Further supporting the notion that human interaction in the NICU is important, we found that an increased number of words spoken around the infant was associated with less infant stress, as measured both physiologically (i.e., changes in vital signs and color changes) and behaviorally (i.e., yawning, gagging, grimacing, finger splaying, or staring). This is a meaningful finding that identifies the important role of human speech in reducing the stressful experiences of the high-risk infant in the NICU. Our findings are consistent with previous reports, which demonstrated relationships between early language exposure and conversational turns, autonomic functioning, and neurodevelopmental outcomes later in infancy and childhood [55]. Other studies have indicated that language exposure, regardless of who provides it, can improve outcomes [35, 62, 63]. Adding language exposure into the NICU experience, through reading programs and enhanced communication, could potentially help close the gap in language exposure between preterm infants hospitalized in the NICU and their full-term counterparts, and could be an important component of aiding the infant in coping with the stressful NICU experience.

We also found that more electronic sounds were related to less hypotonia. There is an assumption that less hypotonia is a positive outcome, although the NNNS Hypotonia score may largely identify tonal differences with emerging or absent reﬂexes or behaviors reﬂective of low tone responses. However, the therapeutic benefit of music, when aligned with the developmental stage of the infant in terms of quality, timing, and intensity, is emerging in the literature [64]. As electronic sounds identified by the LENA include those such as music, this could explain our findings. However, electronic sounds defined on the LENA also could be from alarms, with electronic sounds co-occurring with higher medical complexity, going against this notion. Due to the current limitations of the LENA device not differentiating between potentially therapeutic music and potentially negative alarms, the current investigation is unable to fully untangle this observation. However, it should not be concluded that potentially noxious electronic noise, such as monitor alarms, is beneficial.

Although some relationships of the sound environment to outcomes were observed, there were many associations that failed to reach significance. One possible explanation is that there are complexities within the auditory environment that cannot be fully appreciated with routine measurement (i.e., the role of meaningful language versus distant language, the readiness of the infant for auditory intervention, the sound intensity across time, concurrent medical interventions and conditions, and other contextual environmental factors). Thus, clinical initiatives may be best when they simultaneously focus on minimizing high environmental and unnatural sound levels while increasing meaningful speech, delivered reciprocally and responsively to the infant’s behavioral signs [65].

This study had several limitations. While relationships were observed between auditory exposures and early neurobehavior, such associations were not observed throughout all subscales. This latter finding could be due to a lack of additional relationships, a lack of statistical power due to small sample size, or limitations in neurobehavioral assessment to detect outcomes at this early developmental stage. Early neurobehavioral assessment at/near term-equivalent age is an early marker of the neurological impact most proximal to the NICU exposure, and it is likely modified through post-discharge experiences for its relationship to childhood outcomes. Although auditory monitoring was captured at multiple time points across the NICU stay, the timing (including PMA at capture) varied and was a snapshot in time rather than being comprehensive of every moment of NICU hospitalization. Changes in auditory exposure across time have previously been reported [44], but this study was not able to fully untangle the potential impact of auditory exposure and time-dependent signaling. Further, this study was not able to adequately control for confounds such as increased auditory exposure among infants on more medical equipment, as well as for more positive sounds among infants whose parents engaged in the NICU more often. Neurobehavioral testing at term-equivalent age was conducted over a range of PMA, close to hospital discharge, and may have provided limited visibility at the more immature PMAs. Finally, the NICU environment is rapidly changing with initiatives to improve parent participation and neuroprotection, so the data may need to be validated in a contemporary cohort of infants in the NICU.

The findings of this study support initiatives that intentionally modify sound levels that are too high, while also avoiding silence and ensuring an enriched environment with language exposure in the NICU. Although relationships between auditory exposure and neurobehavior were identified and signal the importance of the modifiable auditory environment of the NICU, more research is needed to aid in identifying the appropriate therapeutic auditory NICU environment. Future studies examining the timing, nature, and dosing of auditory exposures in the NICU and how these inﬂuence infant development, including in early childhood, along-side medical and care factors, will be important in informing clinical approaches to improve short-term neurological outcomes for VPT infants. In addition, using larger samples in future investigations could enable controlling several of the confounding variables, better isolating the relationships of sound exposure to outcome.

## Figures and Tables

**Table 1.** Sample characteristics (*n* = 64 unless otherwise stated).

Parent/family factors	*N* (%) for categorical variables or Mean (SD) for continuous variables
Maternal marital status, married	27 (42%)
Maternal prenatal smoking	13 (20%)
Maternal prenatal use of alcohol	0 (0%)
Maternal prenatal illicit drug use	4 (6%)
Infant race (African-American/Black)	34 (53%)
Insurance type, public (*n* = 46)	28 (61%)
Maternal age	26.9 (5.6)
**Infant clinical factors**	
Infant sex, female	25 (39%)
Delivery, Cesarean	37 (58%)
Brain injury (IVH grade III or IV)	12 (19%)
PDA (*n* = 63)	44 (70%)
NEC (*n* = 61)	12 (20%)
ROP (*n* = 61)	13 (21%)
NICU room type, single-patient room	30 (47%)
EGA	25.5 (1.4)
Birth weight	830.6 (191.8)
Occipital-frontal circumference at birth	23.2 (1.7)
Apgar, 1 min	3.5 (2.3)
Apgar, 5 min	5.8 (1.8)
CRIB score	5.5 (3.7)
Length of stay	102.9 (52.1)
# days on TPN	28.4 (28.1)
Total # of days on breast milk	47.5 (33.8)
# of HFOV days	3.1 (4.7)
# of days with endotracheal intubation	20.9 (27.6)
# of CPAP days	49.4 (225.9)
# of Nasal cannula days	31.5 (65.8)
Total oxygen hours	2213.3 (1302.5)

*IVH* intraventricular hemorrhage, *PDA* patent ductus arteriosus, *NEC* necrotizing enterocolitis, *ROP* retinopathy of prematurity, *EGA* estimated gestational age, *CRIB* Clinical Risk Index for Babies, *TPN* total parenteral nutrition, *HFOV* high-frequency oscillatory ventilation, *CPAP* continuous positive airway pressure.

**Table 2.** Average sound at the NICU bedside.

Sound descriptives (*N* = 62)			
	*Mean*	*Std. Deviation*	*Range*
Meaningful language, in minutes over a 16-h period	13.7	9.7	(0.1–48.9)
Distant language, in minutes over a 16-h period	68.5	45.9	(15.0–319.6)
Electronic sounds, in minutes over a 16-h period	105.2	82.0	(1.6–363.6)
Silence, in minutes over a 16-h period	385.2	232.6	(31.1–905.6)
Noise, in minutes over a 16-h period	375.1	205.3	(7.6–764.1)
Mean peak sound level, in dB, over a 16-h period	86.8	0.9	(84.6–88.2)
Mean average sound level, in dB, over a 16-h period	59.3	2.7	(55.1–68.9)
Adult word count, over a 16-h period	1762.2	1388.7	(6.0–7388.0)

*dB* decibels.

Average sound exposures from 4 separate 16-h recordings within 2 weeks of birth, at 30 weeks postmenstrual age (PMA), 34 weeks PMA, and 35–41 weeks PMA.

**Table 3.** Outcomes at 35–41 weeks postmenstrual age.

Outcome scores descriptives				
	*Mean*	*Std. Deviation*	*Range*	*Comparator Value for Full-Term Healthy Infants* (*Mean* ± SD)
35–41 weeks postmenstrual age				
NNNS Orientation (*N* = 38)	3.61	0.99	(1.67–6.25)	5.30 ± 1.04
Handling (*N* = 41)	0.63	0.22	(0.00–1.00)	0.27 ± 0.27
Quality of Movement (*N* = 44)	3.65	0.84	(2.00–5.67)	3.81 ± 0.78
Self-regulation (*N* = 43)	4.53	0.70	(2.80–6.15)	5.00 ± 0.82
Sub-optimal Reﬂexes (*N* = 44)	7.70	2.21	(2–12)	4.32 ± 1.73
Stress (*N* = 44)	0.32	0.09	(0.08–0.53)	0.15 ± 0.05
Arousal (*N* = 44)	3.81	0.96	(2.14–6.00)	4.16 ± 0.81
Hypertonia (*N* = 44)	1.55	1.37	(0–5)	0.07 ± 0.26
Hypotonia (*N* = 44)	0.91	1.10	(0–5)	0.55 ± 0.76
Asymmetry (*N* = 44)	2.64	2.00	(0–9)	1.93 ± 1.33
Excitability (*N* = 44)	4.57	2.75	(0–11)	4.23 ± 2.10
Lethargy (*N* = 44)	7.02	2.97	(0–13)	6.32 ± 3.24

NICU Network Neurobehavioral Scale (*NNNS)* values for this VPT cohort, with values next to it as a comparator for a 1–2-day-old full-term and health cohort [43].

**Table 4.** Relationships of auditory exposures to outcomes at term age.

Sensory Exposure	Relationship with Outcome	Unstandardized β	*P-value
Electronic sounds	NNNS Hypotonia	−0.003	**0.047**
Silence	NNNS Hypertonia	0.001	**0.010**
Peak Db	NNNS Hypertonia	0.37	**0.040**
Average Db	NNNS Orientation	−0.33	**0.037**
Adult word count	NNNS Stress	−1.36	**0.045**

* *P*-values are from investigating sound exposure area under the curve (AUC) values and outcome using linear regression models while controlling for postmenstrual age at the time of assessment. Only significant values (*p* < 0.05) are included in the table.

*NNNS* NICU Network Neurobehavioral Scales, *dB* decibels.

## Data Availability

Data will be made available to other researchers upon reasonable request and with appropriate documents and agreements in place.
